# Genetic Regulation of Guanylate-Binding Proteins 2b and 5 during Leishmaniasis in Mice

**DOI:** 10.3389/fimmu.2018.00130

**Published:** 2018-02-07

**Authors:** Yahya Sohrabi, Valeryia Volkova, Tatyana Kobets, Helena Havelková, Imtissal Krayem, Martina Slapničková, Peter Demant, Marie Lipoldová

**Affiliations:** ^1^Laboratory of Molecular and Cellular Immunology, Institute of Molecular Genetics of the Czech Academy of Sciences, Prague, Czechia; ^2^Department of Molecular and Cellular Biology, Roswell Park Cancer Institute, Buffalo, NY, United States

**Keywords:** *Leishmania major*, recombinant congenic strains, guanylate-binding proteins, a hidden inflammation, genetic control

## Abstract

Interferon-induced GTPases [guanylate-binding proteins (GBPs)] play an important role in inflammasome activation and mediate innate resistance to many intracellular pathogens, but little is known about their role in leishmaniasis. We therefore studied expression of *Gbp2b*/*Gbp1* and *Gbp5* mRNA in skin, inguinal lymph nodes, spleen, and liver after *Leishmania major* infection and in uninfected controls. We used two different groups of related mouse strains: BALB/c, STS, and CcS-5, CcS-16, and CcS-20 that carry different combinations of BALB/c and STS genomes, and strains O20, C57BL/10 (B10) and B10.O20, OcB-9, and OcB-43 carrying different combinations of O20 and B10 genomes. The strains were classified on the basis of size and number of infection-induced skin lesions as highly susceptible (BALB/c, CcS-16), susceptible (B10.O20), intermediate (CcS-20), and resistant (STS, O20, B10, OcB-9, OcB-43). Some uninfected strains differed in expression of *Gbp2b*/*Gbp1* and *Gbp5*, especially of *Gbp2b*/*Gbp1* in skin. Uninfected BALB/c and STS did not differ in their expression, but in CcS-5, CcS-16, and CcS-20, which all carry BALB/c-derived *Gbp* gene-cluster, expression of *Gbp2b*/*Gbp1* exceeds that of both parents. These data indicate *trans*-regulation of *Gbp*s. Infection resulted in approximately 10× upregulation of *Gbp2b*/*Gbp1* and *Gbp5* mRNAs in organs of both susceptible and resistant strains, which was most pronounced in skin. CcS-20 expressed higher level of *Gbp2b*/*Gbp1* than both parental strains in skin, whereas CcS-16 expressed higher level of *Gbp2b*/*Gbp1* than both parental strains in skin and liver. This indicates a *trans*-regulation present in infected mice CcS-16 and CcS-20. Immunostaining of skin of five strains revealed in resistant and intermediate strains STS, CcS-5, O20, and CcS-20 tight co-localization of Gbp2b/Gbp1 protein with most *L. major* parasites, whereas in the highly susceptible strain, BALB/c most parasites did not associate with Gbp2b/Gbp1. In conclusion, expression of *Gbp2b*/*Gbp1* and *Gbp5* was increased even in organs of clinically asymptomatic resistant mice. It suggests a hidden inflammation, which might contribute to control of persisting parasites. This is supported by the co-localization of Gbpb2/Gbp1 protein and *L. major* parasites in skin of resistant and intermediate but not highly susceptible mice.

## Introduction

Guanylate-binding proteins (GBPs) are components of cell-autonomous immunity playing a key role in response to intracellular infections [reviewed in Ref. (1–3)]. Besides their role in defense against pathogens, they influence cellular proliferation, adhesion, and migration [reviewed in Ref. (4)], and some members have direct anti-tumorigenic effect on tumor cells (5). GBPs and Gbps were first detected as a 67 kDa protein fraction after stimulation of different human and mouse cell lines with IFN (6) and further characterized as a GBP after stimulation of human and mouse fibroblasts with IFNα, IFNβ, and IFNγ (7). There are currently seven GBPs known in humans (encoded by genes located on the chromosome 1) [reviewed in Ref. (3, 8)] and 11 Gbps in mouse. *Gbp2b*/*Gbp1, Gbp2, Gbp3, Gbp5*, and *Gbp7* map to chromosome 3, whereas *Gbp4, Gbp6, Gbp8, Gbp9, Gbp10*, and *Gbp11* are localized on chromosome 5 (9). These proteins are highly conserved and belong to dynamin superfamily—multidomain mechano-chemical GTPases, which are implicated in nucleotide-dependent membrane remodeling events (10, 11).

Guanylate-binding proteins consist of an N-terminal α, β globular large GTPase domain and a α-helical finger-like C-terminal regulatory domain. The domains are connected by a short intermediate region consisting of one α-helix and a short two-stranded β-sheet (12, 13). A GTPase-domain binds guanine nucleotides with low affinities. This induces nucleotide dependent GBP multimerization and hydrolysis of GTP via GDP to GMP [reviewed in Ref. (3)]. Human GBP1, GBP2, and GBP5 and murine Gbp2b/Gbp1, Gbp2, and Gbp5 have at the C-terminus a CaaX sequence (C—cysteine, aa two amino acids, X—terminal amino acid), which directs isoprenylation—the addition of lipid moiety to the protein, which targets proteins to intracellular membranes and facilitates protein-protein interaction (4). Recruitment of proteins to parasitophorous vacuoles harboring pathogens can lead to restriction of pathogen proliferation (14).

GBPs are involved in regulation of inflammasomes—a high-molecular-weight complexes present in the cytosol of stimulated immune cells that mediate the activation of inflammatory caspases resulting in pathogen clearance and/or death of infected cell [reviewed in Ref. (1, 3, 15)]. Gbps can also attack parasites directly via supramolecular complexes (16) and interfere with virus replication (17) or virion assembly (18). Type of effective defense depends on pathogen involved.

A wide range of studies revealed an important role of GBPs in response to different infections including viral (17–20), bacterial (21–24), and protozoan pathogens (14, 16, 25), both vacuolar (14, 16, 21, 24, 25) and cytosolic (17–20).

For example, in human GBP1 influences resistance to vesicular stomatitis virus (19), encephalomyelocarditis virus (19), influenza A viruses (17), and *Chlamydia trachomatis* (22), GBP3 reduces virus titers of influenza A viruses (17) and GBP5 prevents processing and incorporation of the viral glycoprotein Env of HIV-1 (18).

Murine Gbp2b/Gbp1 plays role in defense against *Listeria monocytogenes* and *Mycobacterium bovis* BCG (23), Gbp2 inhibits replication of vesicular stomatitis virus and encephalomyelocarditis virus (20), *Toxoplasma gondii* (14), and *Salmonella typhimurium* (24), and Gbp5 protects against *S. typhimurium* (21) and *M. bovis* BCG (23). Moreover, several Gbps can cooperate for more effective defense. Gene specific-silencing using siRNA established that murine Gbp2b/Gbp1, Gbp5, Gbp7, and Gbp6/10 protect against *M. bovis* BCG and *L. monocytogenes*. A combination of siRNAs exacerbated the loss of function, which indicated that protective Gbps functioned cooperatively (23). Similarly, mutual molecular interactions of murine Gbp2b/Gbp1, Gbp2, Gbp3, Gbp5, and Gbp6 protected against *T. gondii* (16).

*Leishmania* is an obligatory intracellular mammalian pathogen that enters skin by the bite of female phlebotomine sand flies and infects so-called professional phagocytes (neutrophils, monocytes, and macrophages), as well as dendritic cells and fibroblasts. The major host cell is the macrophage where parasites reside inside parasitophorous vacuole, multiply, eventually rupturing the cell and spread to uninfected cells. Infected cells can spread to lymph nodes, spleen, liver, bone marrow, and sometimes lungs [reviewed in Ref. (26)]. The infection can remain asymptomatic or result in one of three main clinical syndromes: the cutaneous form of the disease in dermis, which can be localized or diffuse; mucocutaneous leishmaniasis in the mucosa and the visceral leishmaniasis characterized by splenomegaly and hepatomegaly that results from the metastatic spread of infection to the spleen and liver (27, 28). Manifestations of the disease depend on the infecting species, environmental and social factors, and the genotype of the mammalian host [reviewed in Ref. (26)].

There is very little known about a possible role of GBPs in *Leishmania* infection. Analysis of global gene expression of bone marrow derived macrophages from BALB/c mouse demonstrated upregulation of expression of *Gbp2b/Gbp1, Gbp2, Gbp3, Gbp6*, and *Gbp7* after 24 hours of infection with *Leishmania major* promastigotes (29). Dendritic cells generated from blood of healthy human donors exhibited increased expression of *GBP1* and *GBP2* 16 hours after infection by *L. major* promastigotes, whereas dendritic cells infected by *Leishmania donovani* had increased expression of *GBP1* (30). Comparison of global gene expression in skin lesions of *Leishmania braziliensis*-infected patients with skin of normal skin biopsies revealed upregulation of *GBP5* mRNA (31).

For our analysis, we selected two murine *Gbps* with the C-terminal CaaX sequence enabling targeting proteins to parasitophorous membranes (4). We studied expression of *Gbp2b/Gbp1* and *Gbp5 in vivo* before and 8 weeks after *L. major* infection in 10 mouse strains from two genetically distant but internally related groups: CcS/Dem (BALB/c, STS, CcS-5, CcS-16, CcS-20) and OcB/Dem (O20, C57BL/10 (B10), C57BL/10-*H2^pz^* (B10.O20), OcB-9, OcB-43). Each CcS/Dem strain contains a different, random set of approximately 12.5% genes of the donor strain STS and approximately 87.5% genes of the background strain BALB/c (32, 33). OcB/Dem strains were derived from strains B10.O20 and O20. Strains OcB-43 and OcB-9 contain different 4 and 12.5% of B10 genome on O20 background, respectively; strain B10.O20 contains 4% of O20 genome on B10 background (32, 33). The limited and defined genetic differences between strains in each group (33) make it possible to identify the differences in *Gbp* expression that are controlled by genes outside the *Gbp* coding gene-complex on chromosome 3. Incidence and size of skin lesions indicate that BALB/c and CcS-16 are highly susceptible and B10.O20 is susceptible to *L. major*; whereas CcS-20 is intermediate and STS, CcS-5, O20, B10, OcB-9, and OcB-43 are resistant to this parasite (34) (this study).

We found that the levels of *Gbp2b/Gbp1* and *Gbp5* mRNAs are influenced by *L. major* infection and by genome of the host. The infection caused a large increase of *Gbp2b/Gbp1* and *Gbp5* expression, but *Gbp*s levels in both uninfected and infected mice differ among mouse strains indicating influence of genetic factors. These genetic influences are different in uninfected and infected mice and in some strains there is a clear evidence for a regulation by genes other than the *Gbp* genes (*trans*-regulation). We also show that Gbp2b/Gbp1 protein and *L. major* parasites co-localize in resistant strains STS, CcS-5, and O20 and in the intermediate strain CcS-20 but not in the highly susceptible strain BALB/c.

## Materials and Methods

### Mice

#### mRNA Expression Experiments

A total of 275 (152 infected and 123 uninfected) female mice of strains BALB/c (33 infected and 22 uninfected), STS (20 infected and 13 uninfected), CcS-5 (11 infected and 10 uninfected), CcS-16 (10 infected and 11 uninfected), CcS-20 (12 infected and 12 uninfected), O20/A (O20) (12 infected and 12 uninfected), C57BL/10Sn (B10) (17 infected and 10 uninfected), B10.O20/R164/Dem (B10.O20) (17 infected and 12 uninfected), OcB-9 (7 infected and 7 uninfected), and OcB-43 (13 infected and 14 uninfected) were tested in 15 independent experiments. The age of mice was 8–23 weeks (mean = 11.9 weeks, median = 11 weeks) at the time of infection (start of experiment in control mice). A total of 81 infected mice of strains BALB/c (*n* = 9), STS (*n* = 10), CcS-5 (*n* = 11), O20 (*n* = 12), B10 (*n* = 16), B10.O20 (*n* = 16), and OcB-9 (*n* = 7) from these experiments were used for estimation of parasite load in skin and/or spleen. 40 infected female mice of the strains BALB/c (*n* = 5), STS (*n* = 10), CcS-5 (*n* = 4), CcS-16 (*n* = 12), and CcS-20 (*n* = 9) from additional four experiments were also used for the estimation of parasite load in skin and/or spleen.

#### Immunohistochemistry Experiments

97 (48 infected and 49 uninfected) female mice of strain BALB/c (9 infected and 9 uninfected), STS (9 infected and 9 uninfected), CcS-5 (8 infected and 8 uninfected), CcS-20 (11 infected and 11 uninfected), and O20 (11 infected and 12 uninfected) were tested in two independent experiments. The age of mice was 8–18 weeks (mean 13 weeks, median 14 weeks) at the time of infection.

### Ethics Statement

All experimental protocols utilized in this study comply with the Czech Government Requirements under the Policy of Animal Protection Law (No. 246/1992) and with the regulations of the Ministry of Agriculture of the Czech Republic (No. 207/2004), which are in agreement with all relevant European Union guidelines for work with animals and were approved by the Institutional Animal Care Committee of the Institute of Molecular Genetics AS CR and by Departmental Expert Committee for the Approval of Projects of Experiments on Animals of the Academy of Sciences of the Czech Republic (permissions Nr. 190/2010; 232/2012).

### Parasite

*Leishmania major* LV 561 (MHOM/IL/67/LRCL 137 JERICHO II) was maintained in rump lesions of BALB/c females. Amastigotes were transformed to promastigotes using SNB-9 (35). 10^7^ promastigotes from the passage two cultivated for 6 days were inoculated in 50 µl sterile saline s.c. into mouse rump (36). Control uninfected mice were injected by 50 µl sterile saline.

### Disease Phenotype

The size of the skin lesions was measured every week using the Profi LCD Electronic Digital Caliper Messschieber Schieblehre Messer (Shenzhen Xtension Technology Co., Ltd. Guangdong, China), which has accuracy 0.02 mm. The mice were killed 8 weeks after inoculation. Skin, spleen, liver, and inguinal lymph nodes were collected for later analysis. Preparation of skin samples: approximately 3 mm of border skin surrounding lesion was taken. Hair was removed with scissors. A half of each skin sample was snap-frozen in liquid nitrogen for further RNA and DNA isolations. Another half was fixed in 4% formaldehyde for further paraffin embedding and immunohistochemical analysis. Samples from uninfected mice were obtained from the same rump area and used as a negative control.

### Quantification of Parasite Load by PCR-ELISA

Parasite load was measured in DNA from frozen skin and spleen samples using PCR-ELISA according to the previously published protocol (37). Briefly, total DNA was isolated using a TRI reagent (Molecular Research Center, Cincinnati, OH, USA) standard procedure (https://www.mrcgene.com/wp-content/uploads/2014/06/TRI-LSMarch2017.pdf) or a modified proteinase K procedure (37). For PCR, two primers (digoxigenin-labeled F 5′-ATT TTA CAC CAA CCC CCA GTT-3′ and biotin-labeled R 5′-GTG GGG GAG GGG CGT TCT-3′; VBC Genomics Biosciences Research, Austria) were used for amplification of the 120-bp conservative region of the kinetoplast minicircle of *Leishmania* parasite, and 50 ng of extracted DNA was used per each PCR reaction. For a positive control, 20 ng of *L. major* DNA per reaction was amplified as a highest concentration of standard. A 33-cycle (expression experiments) or 26-cycle (immunostaining experiments) PCR reaction was used for quantification of parasites. Under these conditions, the amount of PCR product is linearly proportional to number of parasites (37). PCR product was measured by the modified ELISA (Pharmingen, San Diego, CA, USA). Concentration of *Leishmania* DNA was determined using the ELISA Reader Tecan and the curve fitter program KIM-E (Schoeller Pharma, Prague, Czech Republic) with least squares-based linear regression analysis.

### RNA Isolation

Mouse spleens, skins, liver, and inguinal lymph nodes were snapped frozen by liquid nitrogen immediately after dissection and stored at −80°C until total RNA extraction. At the time of RNA isolation tissue were homogenized in TRI Reagent (Sigma-Aldrich, Inc., St. Louis, MO, USA) using Polytron PT 2100 homogenizer (Kinematica Ag, Luzern, Switzerland) and immediately followed by total RNA isolation according to the manufacturer’s protocol. RNA concentration was measured with Nanodrop (NanoDrop Technologies, LLC, Wilmington, DL), and quality of RNA was estimated also using Agilent 2100 Bioanalyzer (Agilent Technologies, Inc., Santa Clara, CA, USA). The isolated RNA was stored at −80°C.

### Real-time PCR

One microgram of total RNA was diluted in 8 µl of sterile RNase- and DNase-free water, was treated with 1 µl DNase I (1 U/μl) and 1 µl DNase I reaction buffer (Promega Corporation, Madison, WI, USA), and used for subsequent reverse transcription. Single-strand cDNA was prepared from total RNA using Promega first-strand synthesis method. DNase I-treated RNA was incubated for 10 minutes at 65°C, then cooled quickly on ice for 5 minutes, and then treated with 1 µl DNase I stop solution (Promega Corporation, Madison, WI, USA). For the next step, a mixture containing 1 µl of random hexamers primers (100 ng/1 µl) (Invitrogen, Carlsbad, CA, USA), 5 µl (50 ng/µl) of dNTP mix (Invitrogen, Carlsbad, CA, USA), 5 µl of the reaction buffer (Promega Corporation, Madison, WI, USA), 2.5 µl of RNase/DNase-free water (Invitrogen, Carlsbad, CA, USA), and 0.5 µl of M-MLV Reverse Transcriptase RNAase H Minus Point Mutant (100 U/1 μl) (Promega Corporation, Madison, WI, USA) was added and followed by 60 minutes at 37°C. Single-strand cDNA was kept at −80°C until RT-PCR analysis. Real-time PCR was performed using a BioRad iQ iCycler Detection System (Bio-Rad Laboratories, Inc., Hercules, CA, USA). Primers were designed using Roche Universal ProbeLibrary, ProbeFinder version 2.45 for mouse (*Gbp2b*/*Gbp1*-F AAACCAGGAGGCTACTACCTTTTT, *Gbp2b/Gbp1*-R GTATTTTCTCAGCATCACTTCAGC; *Gbp5*-F TTCACCCAATCTAAGACCAAGAC, *Gbp5*-R AGCACCAGGCTTTCTAGACG; *Gapdh*-F AGAACATCATCCCTGCATCC, *Gapdh*-R ACATTGGGGGTAGGAACAC). Reaction was performed in total volume of 25 µl, including 12.5 µl of 2× SYBR Green Supermix (Bio-Rad Laboratories, Inc., Hercules, CA, USA), 1 µl of each primer of *Gbp2b*/*Gbp1* and *Gbp5* genes (final concentration 6.6 µM), 7.5 µl of water (Invitrogen, Carlsbad, CA, USA), and 3 µl of the cDNA template. Six different samples from each experimental group were used, and all samples were tested in triplets. The average Ct values (cycle threshold) were used for quantification, and the relative amount of each mRNA was normalized to the housekeeping gene, *Gapdh* mRNA. Using the delta Ct value, relative expression was calculated [ratio (reference/target) = 2 Ct (reference) − Ct (target)] × 10,000. All experiments included negative controls containing water instead of cDNA.

### Genotyping of *Gbp* Cluster in OcB Series

DNA was isolated from tails using a standard proteinase procedure. Strains O20, B10, B10.O20, OcB-9, and OcB-43 were genotyped using microsatellite markers D3Mit160 (size of B10 allele 137 bp, size of O20 allele 127 bp) and D3Mit17 (B10 allele 200 bp, O20 allele 174 bp) (Generi Biotech, Hradec Králové, Czech Republic): The DNA genotyping by PCR was performed as described elsewhere (38).

### Immunostaining

After deparaffinization and rehydration, the 3 µm thick slices of skin tissue were 15 minutes heat induced in Tris-EDTA buffer (10 mM Tris, 1 mM EDTA, pH 8.5) for antigen retrieval. For fluorescent labeling of *Leishmania* parasite was used anti-*Leishmania* lipophosphoglycan mouse monoclonal antibody (cat. no. CLP003A; Cedarlane, Hornby, Canada) and TRITC-labeled IgM (115-025-020; Jackson ImmunoResearch, West Grove, PA) all diluted 1:500. Gbp2b/Gbp1 protein was stained with rabbit anti-Gbp1 Polyclonal antibody (PA5-23509; Thermo Fisher Scientific, Rockford, IL, USA) diluted 1:100 and anti-rabbit-AlexaFluor-647 (cat. no. 711-605-152; Jackson ImmunoResearch, West Grove, PA) diluted 1:500. Nuclei of the cells were stained with bisBenzimide H33258 (Sigma-Aldrich, St. Louis, MO, USA) 10 mg per 1 ml diluted 1:1,000. Images were captured with microscope Leica DM6000 objective HCX PL Apo 40×/0.75 PH2 and color camera Leica DFC490. Evaluation of images was done with Fiji ImageJ 1.51n. 10 fields (320.66 × 239.57 µM) from each mouse were analyzed.

### Statistical Analysis

The differences among strains within each of the two groups in expression of *Gbp2b*/*Gbp1* and *Gbp5* and the differences between uninfected and infected mice were evaluated by Mann–Whitney test using the program Statistica for Windows 12.0 (StatSoft, Inc., Tulsa, OK, USA). The results were corrected for multiple testing by Bonferroni correction. The correction factor was 10× both for intragroup differences and differences between infected and uninfected mice of the same strain.

## Results

### Mouse Strains Differ in Expression of Both *Gbp2b*/*Gbp1* and *Gbp5* in Uninfected Mice

**Figure 1:**
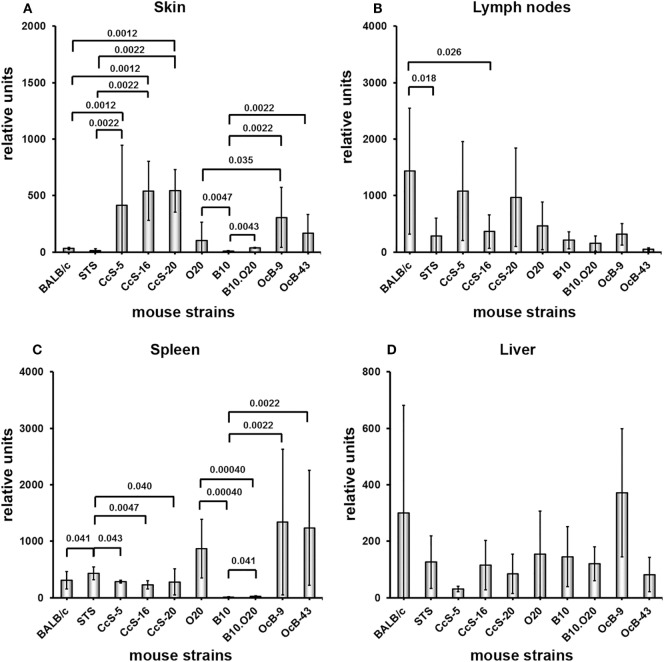
Differences in expression of *Gbp2b*/*Gbp1* in organs of uninfected mice. Expression of *Gbp2b*/*Gbp1* in skin **(A)**, lymph nodes **(B)**, spleen **(C)**, and liver **(D)** of uninfected female mice of strains BALB/c (*n* = 7 skin, 9 lymph nodes, 11 spleen, 9 liver), STS (6 skin, 9 lymph nodes, 8 spleen, 6 liver), CcS-5 (6 skin, 6 lymph nodes, 6 spleen, 6 liver), CcS-16 (6 skin, 6 lymph nodes, 6 spleen, 6 liver), CcS-20 (6 skin, 6 lymph nodes, 7 spleen, 6 liver), O20 (7 skin, 6 lymph nodes, 9 spleen, 6 liver), B10 (6 skin, 7 lymph nodes, 6 spleen, 6 liver), B10.O20 (5 skin, 7 lymph nodes, 6 spleen, 8 liver), OcB-9 (6 skin, 6 lymph nodes, 6 spleen, 5 liver), and OcB-43 (6 skin, 6 lymph nodes, 6 spleen, 7 liver) were compared. The data show the means ± SD. Only the differences between parental strains BALB/c and STS and strains of CcS/Dem series and parental strains O20 and B10 and strains of OcB/Dem series are shown. Nominal *P* values are shown.

**Figure 2:**
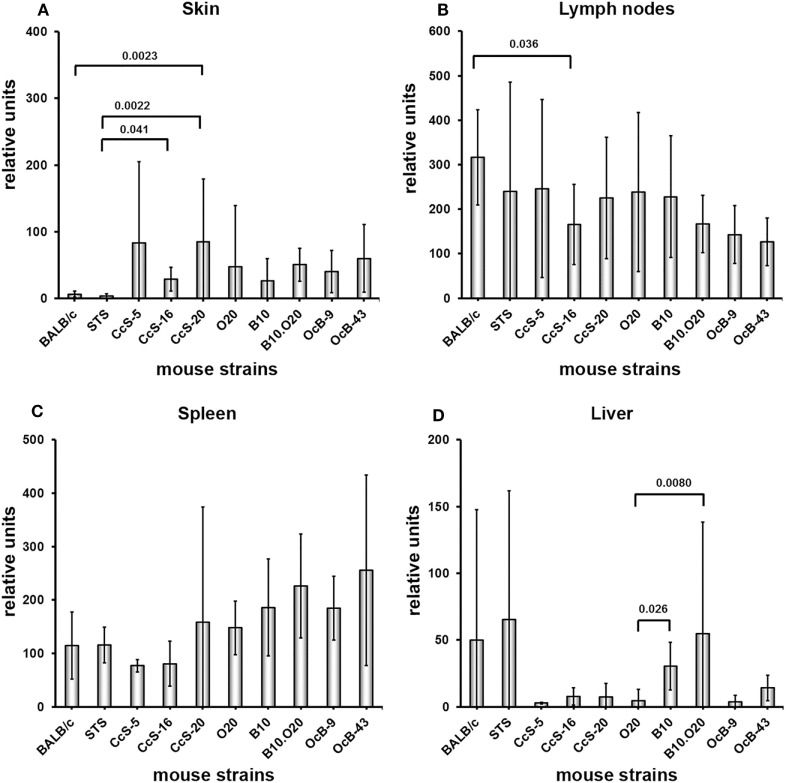
Differences in expression of *Gbp5* in organs of uninfected mice. Expression of *Gbp5* in skin **(A)**, lymph nodes **(B)**, spleen **(C)** and liver **(D)** of uninfected female mice of strains of strains BALB/c (*n* = 7 skin, 9 lymph nodes, 11 spleen, 9 liver), STS (6 skin, 9 lymph nodes, 8 spleen, 6 liver), CcS-5 (6 skin, 6 lymph nodes, 6 spleen, 6 liver), CcS-16 (6 skin, 6 lymph nodes, 6 spleen, 6 liver), CcS-20 (6 skin, 6 lymph nodes, 7 spleen, 6 liver), O20 (7 skin, 6 lymph nodes, 9 spleen, 6 liver), B10 (6 skin, 7 lymph nodes, 6 spleen, 6 liver), B10.O20 (5 skin, 7 lymph nodes, 6 spleen, 8 liver), OcB-9 (6 skin, 6 lymph nodes, 6 spleen, 5 liver), and OcB-43 (6 skin, 6 lymph nodes, 6 spleen, 7 liver) were compared. The data show the means ± SD. Only the differences between parental strains BALB/c and STS and strains of CcS/Dem series and parental strains O20 and B10 and strains of OcB/Dem series are shown. Nominal *P* values are shown.

We observed strong genetic influence on mRNA levels of tested *Gbp*s. We have examined expression of *Gbp2b*/*Gbp1* (Figure 1) and *Gbp5* (Figure 2) in skin, inguinal lymph nodes, spleen, and liver of uninfected mice belonging to two genetically different series of strains—CcS/Dem (BALB/c, STS, CcS-5, CcS-16, CcS-20) and OcB/Dem (O20, B10, B10.O20, OcB-9, OcB-43). We have compared expression in parental strains BALB/c and STS with the strains of CcS/Dem series, and expression in parental strains O20 and B10 with the strains of OcB/Dem series (Figures 1 and 2), as well as expression of the strains within CcS/Dem and OcB/Dem series in skin (Tables 1A,C), lymph nodes (Tables 2A,C), spleen (Tables 3A,C), and liver (Tables 4A,C).

**Table 1:** Comparison of expression of *Gbp2b*/*Gbp1* or *Gbp5* among mouse strains of CcS/Dem and OcB/Dem series in skin.

CcS/Dem series					OcB/Dem series				
Strain	BALB/c	STS	CcS-5	CcS-16	Strain	O20	B10	B10.O20	OcB-9
**A. *Gbp2b*/*Gbp1* uninfected**									

**BALB/c**					**O20**				
**STS**	0.11				**B10**	0.0047			
**CcS-5**	0.0012	0.0022			**B10.O20**	1	0.0043		
**CcS-16**	0.0012	0.0022	0.24		**OcB-9**	0.035	0.0022	0.0043	
**CcS-20**	0.0012	0.0022	0.31	0.82	**OcB-43**	0.23	0.0022	0.052	0.18

**B. *Gbp2b*/*Gbp1* infected**									

**BALB/c**					**O20**				
**STS**	0.54				**B10**	0.043			
**CcS-5**	0.86	0.73			**B10.O20**	0.0012	0.19		
**CcS-16**	0.0076	0.0012	0.0022		**OcB-9**	0.54	0.40	0.0047	
**CcS-20**	0.0048	0.0012	0.0022	0.59	**OcB-43**	0.91	0.036	0.00012	0.30

**C. *Gbp5* uninfected**									

**BALB/c**					**O20**				
**STS**	0.37				**B10**	0.73			
**CcS-5**	0.30	0.13			**B10.O20**	0.20	0.25		
**CcS-16**	0.051	0.041	0.82		**OcB-9**	0.45	0.39	0.79	
**CcS-20**	0.0023	0.0022	0.31	0.093	**OcB-43**	0.14	0.18	0.79	0.59

**D. *Gbp5* infected**									

**BALB/c**					**O20**				
**STS**	0.54				**B10**	0.83			
**CcS-5**	0.18	0.37			**B10.O20**	0.21	0.076		
**CcS-16**	0.088	0.0012	0.0022		**OcB-9**	0.088	0.15	0.000026	
**CcS-20**	0.036	0.0023	0.0022	0.49	**OcB-43**	0.50	0.24	0.51	0.0012

*A. Differences of Gbp2b/Gbp1 expression in uninfected skin; B. Differences of Gbp2b/Gbp1 expression in skin after 8 weeks of infection; C. Differences of Gbp5 expression in uninfected skin; D. Differences of Gbp5 expression in skin after 8 weeks of infection. Green: nominal (uncorrected) P value < 0.05; red: difference significant after correction for multiple testing at P < 0.05*.

**Table 2:** Comparison of expression of *Gbp2b*/*Gbp1* or *Gbp5* among mouse strains of CcS/Dem and OcB/Dem series in inguinal lymph nodes.

CcS/Dem series					OcB/Dem series				
Strain	BALB/c	STS	CcS-5	CcS-16	Strain	O20	B10	B10.O20	OcB-9
**A. *Gbp2b*/*Gbp1* uninfected**									

**BALB/c**					**O20**				
**STS**	0.018				**B10**	0.53			
**CcS-5**	0.69	0.065			**B10.O20**	0.53	0.62		
**CcS-16**	0.026	0.59	0.18		**OcB-9**	0.70	0.37	0.051	
**CcS-20**	0.61	0.24	1	0.39	**OcB-43**	0.31	0.10	0.035	0.0022

**B. *Gbp2b*/*Gbp1* infected**									

**BALB/c**					**O20**				
**STS**	0.18				**B10**	0.021			
**CcS-5**	0.0049	0.13			**B10.O20**	0.038	0.28		
**CcS-16**	0.40	0.18	0.0022		**OcB-9**	0.30	0.081	0.13	
**CcS-20**	0.96	0.31	0.0087	0.49	**OcB-43**	0.15	0.25	0.019	0.64

**C. *Gbp5* uninfected**									

**BALB/c**					**O20**				
**STS**	0.39				**B10**	0.95			
**CcS-5**	0.61	0.82			**B10.O20**	0.37	0.62		
**CcS-16**	0.036	1	0.59		**OcB-9**	0.24	0.23	0.63	
**CcS-20**	0.22	1	1	0.39	**OcB-43**	0.18	0.051	0.30	0.59

**D. *Gbp5* infected**									

**BALB/c**					**O20**				
**STS**	0.96				**B10**	0.14			
**CcS-5**	0.53	0.59			**B10.O20**	0.37	0.69		
**CcS-16**	0.010	0.31	0.0022		**OcB-9**	0.92	0.24	0.48	
**CcS-20**	0.53	0.94	0.18	0.31	**OcB-43**	0.060	0.44	0.22	0.34

*A. Differences of Gbp2b/Gbp1 expression in uninfected lymph nodes; B. Differences of Gbp2b/Gbp1 expression in lymph nodes after 8 weeks of infection; C. Differences of Gbp5 expression in uninfected lymph nodes; D. Differences of Gbp5 expression in lymph nodes after 8 weeks of infection. Green: nominal (uncorrected) P value < 0.05; red: difference significant after correction for multiple testing at P < 0.05*.

**Table 3:** Comparison of expression of *Gbp2b*/*Gbp1* or *Gbp5* among mouse strains of CcS/Dem and OcB/Dem series in spleen.

CcS/Dem series					OcB/Dem series				
Strain	BALB/c	STS	CcS-5	CcS-16	Strain	O20	B10	B10.O20	OcB-9
**A. *Gbp2b*/*Gbp1* uninfected**									

**BALB/c**					**O20**				
**STS**	0.041				**B10**	0.00040			
**CcS-5**	0.66	0.043			**B10.O20**	0.00040	0.041		
**CcS-16**	0.40	0.0047	0.24		**OcB-9**	0.69	0.0022	0.0022	
**CcS-20**	0.25	0.040	0.23	0.84	**OcB-43**	0.53	0.0022	0.0022	0.94

**B. *Gbp2b*/*Gbp1* infected**									

**BALB/c**					**O20**				
**STS**	0.35				**B10**	0.000022			
**CcS-5**	0.40	0.018			**B10.O20**	0.00080	0.26		
**CcS-16**	0.44	0.018	0.49		**OcB-9**	0.53	0.00025	0.0022	
**CcS-20**	0.22	0.049	0.93	0.54	**OcB-43**	0.96	0.00025	0.0022	0.39

**C. *Gbp5* uninfected**									

**BALB/c**					**O20**				
**STS**	0.55				**B10**	0.46			
**CcS-5**	0.15	0.059			**B10.O20**	0.18	0.70		
**CcS-16**	0.30	0.14	1		**OcB-9**	0.27	1	0.49	
**CcS-20**	0.60	0.19	0.73	0.95	**OcB-43**	0.33	0.94	0.94	0.94

**D. *Gbp5* infected**									

**BALB/c**					**O20**				
**STS**	0.031				**B10**	0.32			
**CcS-5**	0.66	0.032			**B10.O20**	1	0.22		
**CcS-16**	0.08	0.00011	0.0022		**OcB-9**	0.61	0.26	0.59	
**CcS-20**	0.62	0.0061	0.18	0.247	**OcB-43**	0.61	0.26	0.94	0.24

*A. Differences of Gbp2b/Gbp1 expression in uninfected spleen; B. Differences of Gbp2b/Gbp1 expression in spleen after 8 weeks of infection; C. Differences of Gbp5 expression in uninfected spleen; D. Differences of Gbp5 expression in spleen after 8 weeks of infection. Green: nominal (uncorrected) P value < 0.05; red: difference significant after correction for multiple testing at P < 0.05*.

**Table 4:** Comparison of expression of *Gbp2b*/*Gbp1* or *Gbp5* among mouse of CcS/Dem and OcB/Dem series strains in liver.

CcS/Dem series					OcB/Dem series				
Strain	BALB/c	STS	CcS-5	CcS-16	Strain	O20	B10	OcB-9
**A. *Gbp2b*/*Gbp1* uninfected**									

**BALB/c**					**O20**				
**STS**	0.46				**B10**	0.94			
**CcS-5**	0.088	0.24			**B10.O20**	0.85	0.66		
**CcS-16**	0.53	0.82	0.0022		**OcB-9**	0.052	0.13	0.045	
**CcS-20**	0.18	0.39	0.39	0.70	**OcB-43**	0.63	0.45	0.19	0.010

**B. *Gbp2b*/*Gbp1* infected**									

**BALB/c**					**O20**				
**STS**	0.072				**B10**	0.43			
**CcS-5**	0.21	0.31			**B10.O20**	0.66	0.98		
**CcS-16**	0.000074	0.0022	0.0022		**OcB-9**	0.054	0.0018	0.035	
**CcS-20**	0.0085	0.14	0.073	0.0012	**OcB-43**	0.49	0.042	0.40	0.014

**C. *Gbp5* uninfected**									

**BALB/c**					**O20**				
**STS**	0.33				**B10**	0.026			
**CcS-5**	0.86	0.18			**B10.O20**	0.0080	0.85		
**CcS-16**	0.46	0.94	0.041		**OcB-9**	0.79	0.052	0.011	
**CcS-20**	0.86	0.59	0.59	0.49	**OcB-43**	0.073	0.14	0.19	0.030

**D. *Gbp5* infected**									

**BALB/c**					**O20**				
**STS**	0.00030				**B10**	0.069			
**CcS-5**	0.0047	0.31			**B10.O20**	0.026	0.45		
**CcS-16**	0.77	0.0022	0.0022		**OcB-9**	0.78	0.54	0.33	
**CcS-20**	0.011	0.84	0.95	0.0023	**OcB-43**	0.23	0.13	0.062	0.95

*A. Differences of Gbp2b/Gbp1 expression in uninfected liver; B. Differences of Gbp2b/Gbp1 expression in liver after 8 weeks of infection; C. Differences of Gbp5 expression in uninfected liver; D. Differences of Gbp5 expression in liver after 8 weeks of infection. Green: nominal (uncorrected) P value < 0.05; red: difference significant after correction for multiple testing at P < 0.05*.

Expression of *Gbp2b*/*Gbp1* in background strain BALB/c and donor strain STS in skin (Figure 1A; Table 1A) does not differ, whereas strains CcS-5, CcS-16, and CcS-20, each carrying a different set of 12.5% genes of STS on BALB/c background, exhibit higher expression than either parent (Figure 1A; Table 1A). Expression of *Gbp2b*/*Gbp1* in parental strains of OcB/Dem series O20 and B10 in skin differed (*P* = 0.0047); strains B10.O20, OcB-9, and OcB-43 exceeded in *Gbp2b*/*Gbp1* expression of the parental strain B10 but not O20 (Figure 1A; Table 1A).

We have observed differences in the expression of *Gbp2b*/*Gbp1* among mouse strains also in other tested organs (Figures 1B–D; Tables 2A, 3A, and 4A). Strains OcB-43 and OcB-9 differed in the expression of *Gbp2b*/*Gbp1* in lymph nodes (Table 2A), CcS-16 exhibited lower expression than STS in spleen (Figure 2C; Table 3A), B10 and B10.O20 exhibited lowest expression in spleen and differed from strains O20, OcB-9, and OcB-43, CcS-5 exhibited lower expression than CcS-16 in liver (Figure 1D; Table 4A); however, expression in none of the CcS or OcB strains exceeded the range of expression in both parental strains.

Expression of *Gbp5* in skin did not differ in parental strains of CcS/Dem series BALB/c and STS (Figure 2A; Table 1C), and expression of *Gbp5* in CcS-20 exceeded the expression of both parental strains (Figure 2A; Table 1C). There was no difference in *Gbp5* expression among strains of OcB/Dem series (Table 1C).

We did not find any significant differences in expression of *Gbp5* among the strains of both CcS/Dem and OcB/Dem series in the lymph nodes, spleen, and liver; none of the strains in CcS/Dem or in OcB/Dem series differed from either parent (Figures 2B–D; Tables 2C, 3C, and 4C).

### Upregulation of *Gbp2b*/*Gbp1* and *Gbp5* mRNAs after Infection

**Figure 3:**
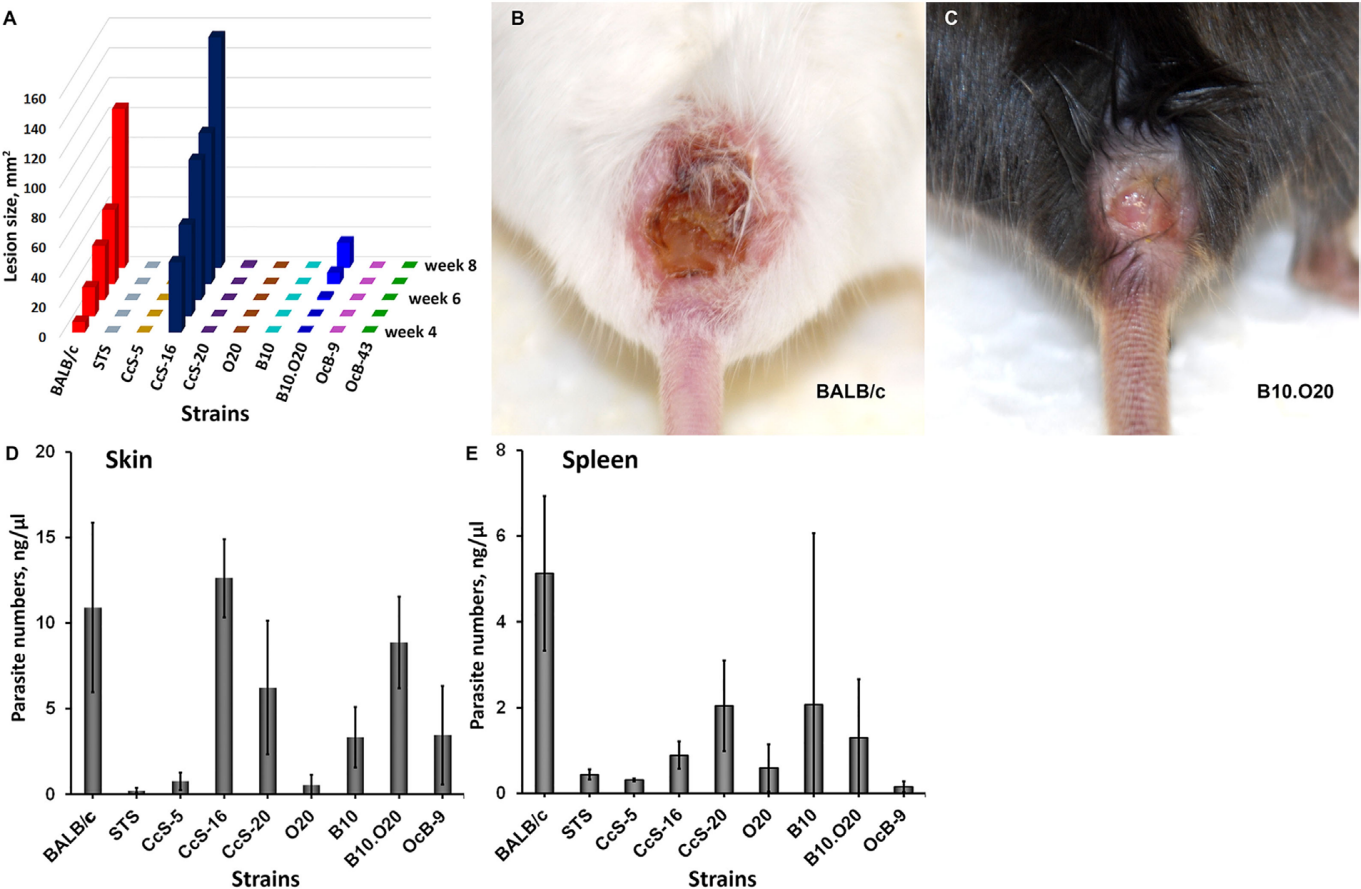
Skin lesion development and parasite load in skin and spleen of infected mice. Kinetics of lesion development from week 4 (appearance of lesions in highly susceptible strains) till week 8 (end of experiment) **(A)**. Median values of skin lesions of mice tested in expression experiments are shown. Skin lesion caused by *Leishmania major* in female mouse of BALB/c strain at week 8 after infection **(B)**. Skin lesion caused by *L. major* in female mouse of B10.O20 strain at week 8 after infection **(C)**. Parasite load in skin **(D)** and spleen **(E)** of infected female mice of strains BALB/c (*n* = 5 skin, 11 spleen), STS (13 skin, 7 spleen), CcS-5 (11 skin, 4 spleen), CcS-16 (6 skin, 6 spleen), CcS-20 (7 skin, 9 spleen), O20 (6 skin, 12 spleen), B10 (9 skin, 13 spleen), B10.O20 (7 skin, 15 spleen), and OcB-9 (7 skin, 7 spleen). The data show the means ± SD.

In susceptible mice, pathology started as a nodule at the site of *L. major* infection appearing between weeks 2 and 4, which progressed in susceptible strains into a skin lesion (Figure 3A) (34). Strains BALB/c and CcS-16 are highly susceptible and develop large skin lesions (Figures 3A,B), B10.O20 develops moderate skin lesions (Figures 3A,C), CcS-20 is intermediate (Figures 3A,D) (34), and STS, CcS-5, O20, B10, OcB-9, and OcB-43 are resistant with no or minimal skin lesions (Figure 3A). All tested strains contain parasites in skin (Figure 3D) and spleen (Figure 3E), although the parasite load in resistant strains STS, CcS-5 and O20 (skin and spleen), and OcB-9 (spleen) is low.

**Figure 4:**
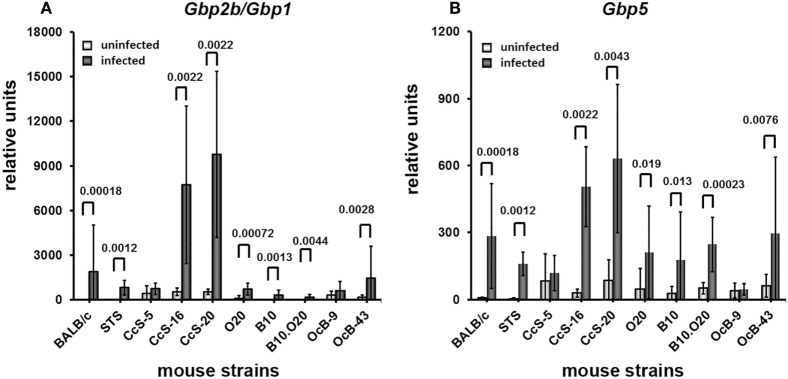
Differences in expression of *Gbp2b*/*Gbp1* and *Gbp5* in skin of uninfected and infected mice. Expression of *Gbp2b/Gbp1* **(A)** and *Gbp5* **(B)** in skin of uninfected and infected female mice of strains BALB/c (9 infected and 7 uninfected), STS (7 infected and 6 uninfected), CcS-5 (6 infected and 6 uninfected), CcS-16 (6 infected and 6 uninfected), CcS-20 (6 infected and 6 uninfected), O20 (10 infected and 7 uninfected), B10 (8 infected and 6 uninfected), B10.O20 (13 infected and 5 uninfected), OcB-9 (7 infected and 6 uninfected), and OcB-43 (9 infected and 6 uninfected) were compared. Animals were subcutaneously inoculated with 10^7^ promastigotes of *Leishmania major*. Control, uninfected mice were kept in the same animal facility. Both groups were killed in the same time—after 8 weeks of infection or start of experiment. The data show the means ± SD. Nominal *P* values are shown.

**Figure 5:**
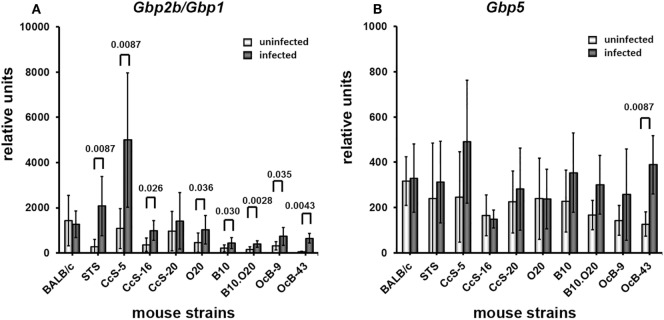
Differences in expression of *Gbp2b*/*Gbp1* and *Gbp5* in inguinal lymph nodes of uninfected and infected mice. Expression of *Gbp2b/Gbp1* **(A)** and *Gbp5* **(B)** in inguinal lymph nodes of uninfected and infected female mice of strains BALB/c (11 infected and 9 uninfected), STS (6 infected and 6 uninfected), CcS-5 (6 infected and 6 uninfected), CcS-16 (6 infected and 6 uninfected), CcS-20 (6 infected and 6 uninfected), O20 (9 infected and 6 uninfected), B10 (13 infected and 7 uninfected), B10.O20 (11 infected and 7 uninfected), OcB-9 (7 infected and 6 uninfected), and OcB-43 (5 infected and 6 uninfected) were compared. Animals were subcutaneously inoculated with 10^7^ promastigotes of *Leishmania major*. Control, uninfected mice were kept in the same animal facility. Both groups were killed after 8 weeks of infection. The data show the means ± SD from 12 independent experiments. Nominal *P* values are shown.

**Figure 6:**
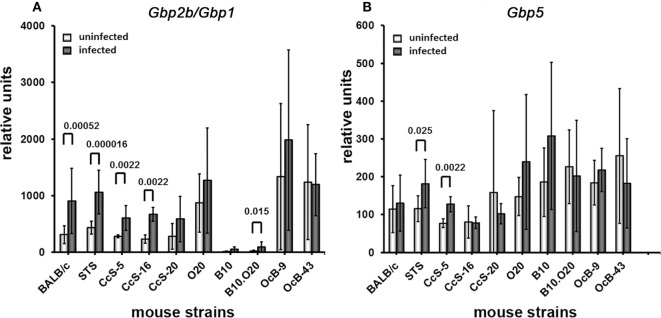
Differences in expression of *Gbp2b*/*Gbp1* and *Gbp5* in spleen of uninfected and infected mice. Expression of *Gbp2b/Gbp1* **(A)** and *Gbp5* **(B)** in spleens of uninfected and infected female mice of strains BALB/c (14 infected and 11 uninfected), STS (12 infected and 8 uninfected), CcS-5 (6 infected and 6 uninfected), CcS-16 (6 infected and 6 uninfected), CcS-20 (5 infected and 7 uninfected), O20 (9 infected and 9 uninfected), B10 (10 infected and 6 uninfected), B10.O20 (6 infected and 6 uninfected), OcB-9 (6 infected and 6 uninfected), and OcB-43 (6 infected and 6 uninfected) were compared. Animals were subcutaneously inoculated with 10^7^ promastigotes of *Leishmania major*. Control, uninfected mice were kept in the same animal facility. Both groups were killed after 8 weeks of infection. The data show the means ± SD from 12 independent experiments. Nominal *P* values are shown.

**Figure 7:**
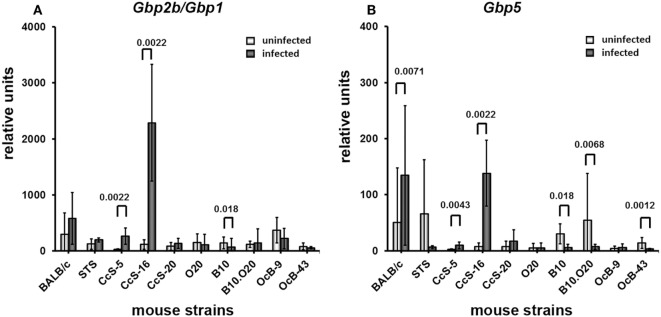
Differences in expression of *Gbp2b*/*Gbp1* and *Gbp5* in liver of uninfected and infected mice. Expression of *Gbp2b/Gbp1* **(A)** and *Gbp5* **(B)** in liver uninfected and infected female mice of strains BALB/c (13 infected and 9 uninfected), STS (6 infected and 6 uninfected), CcS-5 (6 infected and 6 uninfected), CcS-16 (6 infected and 6 uninfected), CcS-20 (7 infected and 6 uninfected), O20 (8 infected and 6 uninfected), B10 (12 infected and 6 uninfected), B10.O20 (11 infected and 8 uninfected), OcB-9 (7 infected and 5 uninfected), and OcB-43 (7 infected and 6 uninfected) were compared. Animals were subcutaneously inoculated with 10^7^ promastigotes of *Leishmania major*. Control, uninfected mice were kept in the same animal facility. Both groups were killed after 8 weeks of infection. The data show the means ± SD from 12 independent experiments. Nominal *P* values are shown.

Infection increased the expression of *Gbp2b*/*Gbp1* and *Gbp5* in organs of tested mice, the highest increase was observed in skin (Figures 4–7). All tested strains except CcS-5 and OcB-9 exhibited significantly higher expression of *Gbp2b*/*Gbp1* and *Gbp5* in skin after infection, irrespective of their susceptibility or resistance status (Figure 4A). Similarly as in uninfected mice, levels of *Gbp2b*/*Gbp1* mRNA in CcS-16 and CcS-20 exceeded those in both parental strains BALB/c and STS (Figure S1A in Supplementary Material; Table 1B); *Gbp5* expression in infected CcS-20 also exceeded that in both BALB/c and STS (Figure S2A in Supplementary Material; Table 1D).

Infection also induced increase of *Gbp2b*/*Gbp1* in inguinal lymph nodes of all strains except BALB/c and CcS-20, the highest expression was observed in CcS-5 (Figure 5A), which differed from all tested strains except STS (Figure S1B in Supplementary Material; Table 2B), but only increase of expression of B10.O20 and OcB-43 was significant after correction for multiple testing; we did not observe significant increase of *Gbp5* mRNA in lymph nodes (Figure 5B).

Four strains (BALB/c, STS, CcS-5, and CcS-16) show significantly increased expression of *Gbp2b*/*Gbp1* in spleen (Figure 6A). Expression of *Gbp5* was increased in CcS-5 (Figure 6B).

In liver, infection induced significant increases of *Gbp2b*/*Gbp1* mRNA in strains of CcS/Dem series, CcS-5, and CcS-16 (Figure 7A). Level of *Gbp2b*/*Gbp1* mRNA in CcS-16 is highest from all tested strains (Figure S1 in Supplementary Material; Table 4B). Expression of *Gbp5* was significantly increased in CcS/Dem strains CcS-5 and CcS-16 and decreased in OcB/Dem strain OcB-43 (Figure 7B; Table 4D).

### Gbp2b/Gbp1 Protein Tends to Co-Localize with *Leishmania* Parasites in Skin of Resistant and Intermediate Strains but Not in the Highly Susceptible Strain BALB/c

**Figure 8:**
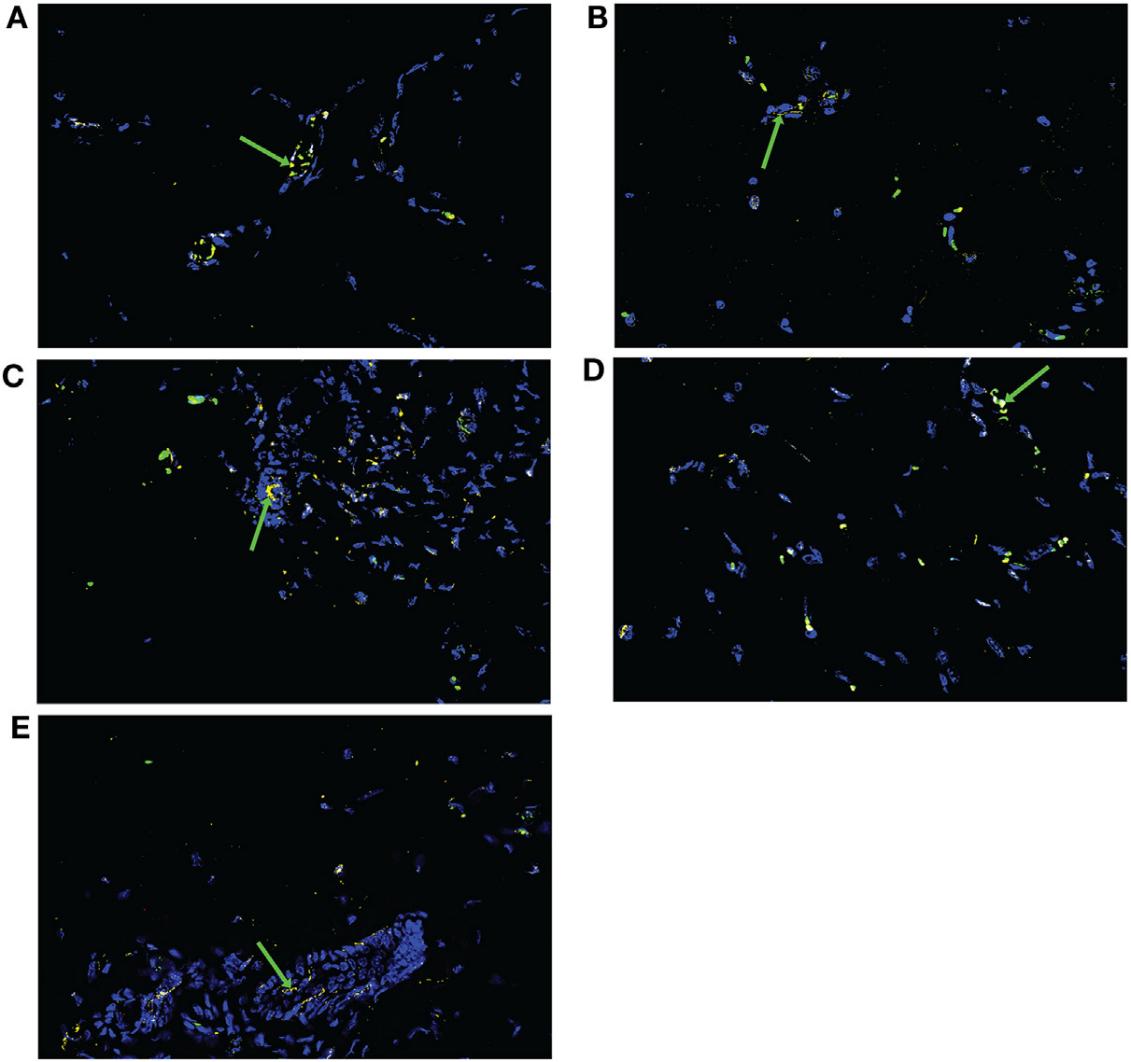
Gbp2b/Gbp1 protein in skin of uninfected mice. Slices of skin tissue of females of BALB/c **(A)**, STS **(B)**, CcS-5 **(C)**, CcS-20 **(D)**, and O20 **(E)** mice were stained with the rabbit anti-Gbp1 Polyclonal antibody (PA5-23509, Thermo Fisher Scientific, Rockford, IL, USA) diluted 1:100 and anti-rabbit-AlexaFluor-647 (cat. no. 711-605-152; Jackson ImmunoResearch, West Grove, PA) diluted 1:500. Nuclei of the cells were stained with bisBenzimide H33258 (Sigma-Aldrich, St. Louis, MO, USA) 10 mg per 1 ml diluted 1:1,000. Images were captured with microscope Leica DM6000 objective HCX PL Apo 40x/0.75 PH2 and color camera Leica DFC490. Evaluation of images was done with Fiji ImageJ 1.51n. Figures are representatives of data from 8 to 12 mice (see Materials and Methods) in 3 of them 10 fields (320.66 × 239.57 µM) from each mouse were analyzed, in the rest one field was analyzed to verify the results. Green arrows show Gbp2b/Gbp1 protein (yellow color), cell nuclei are stained in blue.

**Figure 9:**
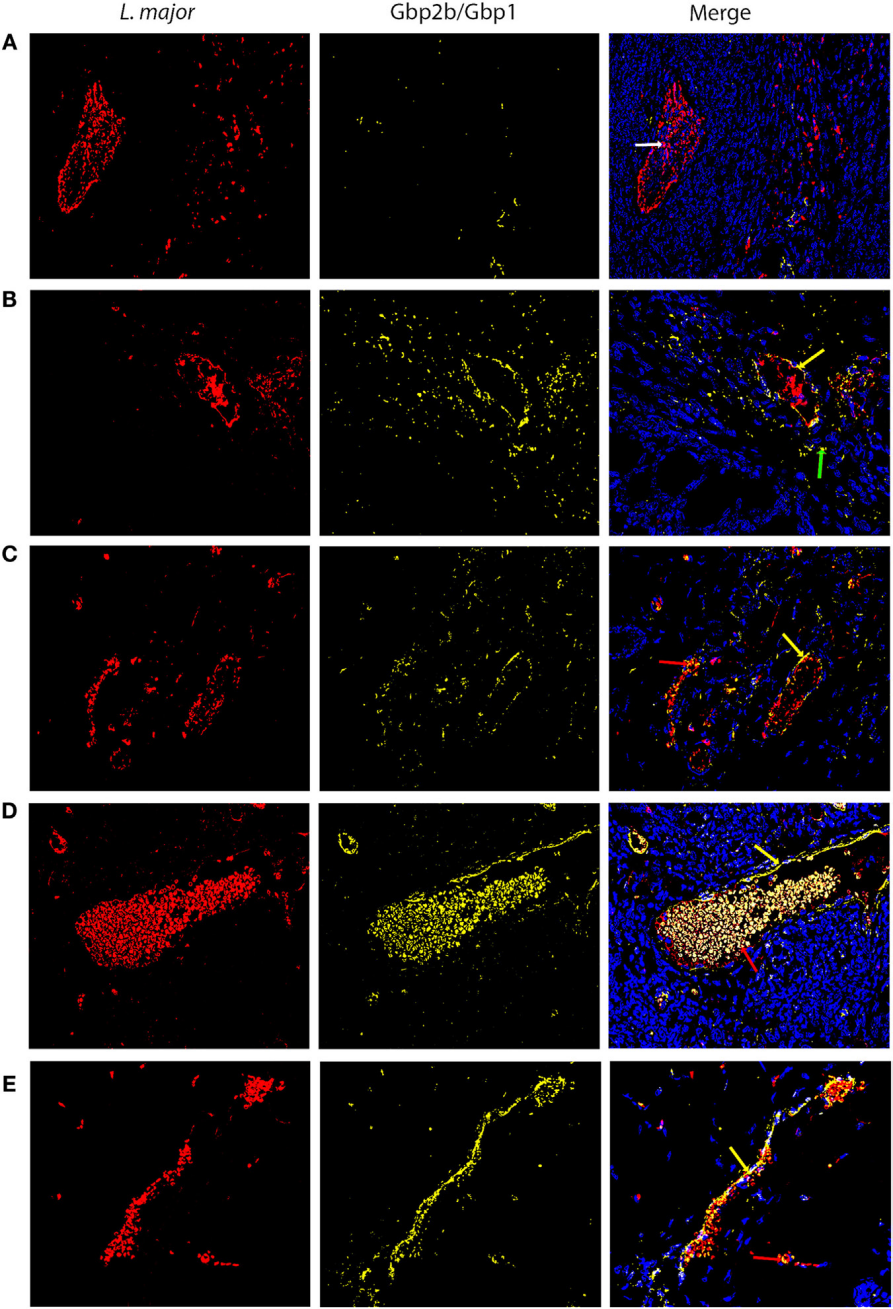
Gbp2b/Gbp1 protein and *Leishmania major* parasites in skin of infected mice. Slices of skin tissue of females of BALB/c **(A)**, STS **(B)**, CcS-5 **(C)**, CcS-20 **(D)**, and O20 **(E)** mice infected for 8 weeks with *L. major* were stained with the anti-*Leishmania* lipophosphoglycan mouse monoclonal antibody (cat. no. CLP003A, Cedarlane, Hornby, Canada) and TRITC labeled IgM (115-025-020, Jackson ImmunoResearch, West Grove, PA) all diluted 1:500 and the rabbit anti-Gbp1 Polyclonal antibody (PA5-23509, Thermo Fisher Scientific, Rockford, IL, USA) diluted 1:100 and anti-rabbit-AlexaFluor-647 (cat. no. 711-605-152; Jackson ImmunoResearch, West Grove, PA) diluted 1:500. Nuclei of the cells were stained with bisBenzimide H33258 (Sigma-Aldrich, St. Louis, MO, USA) 10 mg per 1 ml diluted 1:1,000. Images were captured with microscope Leica DM6000 objective HCX PL Apo 40×/0.75 PH2 and color camera Leica DFC490. Evaluation of images was done with Fiji ImageJ 1.51n. Figures are representatives of data from 8 to 11 mice (see Materials and Methods) in 3 of them 10 fields (320.66 × 239.57 µM) from each mouse were analyzed, in the rest one field was analyzed to verify the results. White arrow shows *L. major* amastigotes (red color), green arrows show Gbp2b/Gbp1 protein (yellow color), red arrows point to amastigotes co-localized with Gbp2b/Gbp1, whereas yellow arrows show either Gbp2b/Gbp1 surrounding parasite clusters or stretch of parasites and Gbp2b/Gbp1. Cell nuclei are stained in blue.

Expression of *Gbp2b*/*Gbp1* mRNA was highest in skin of infected mice (Figure S1 in Supplementary Material; Figure 4), we have therefore analyzed by immunohistochemistry a presence of Gbp2b/Gbp1 protein in the skin of selected strains BALB/c, STS, CcS-5, CcS-20, and O20 and its relationship to *L. major* parasite in infected mice. Figure 8 shows the presence of Gbp2b/Gbp1 protein in the skin of uninfected strains. The comparison of position of *L. major* and Gbp2b/Gbp1 in the skin of chronically infected highly susceptible strain BALB/c showed few Gbp2b/Gbp1 in the vicinity of *L. major* parasites, but a large part of parasites was free of Gbp2b/Gbp1 (Figure 9A); the comparison of parasite load in the skin of the tested strains is shown in Figure S3 in Supplementary Material. In resistant strains STS (Figure 9B), CcS-5 (Figure 9C), and O20 (Figure 9E) and in intermediate strain CcS-20 (Figure 9D), Gbp2b/Gbp1 co-localized with clusters of parasites (Figures 9B–E) that in some places formed large clusters or long stretches. Gbp2b/Gbp1 either surrounded these clusters (Figures 9B–D) or formed stretches consisting of *L. major* parasites and Gbp2b/Gbp1 (Figures 9C,E). The tightest co-localization was observed in strains CcS-20 (Figure 9D) and O20 (Figure 9E).

## Discussion

### Genetic Influence on Expression of *Gbp2b*/*Gbp1* and *Gbp5*

Tested strains exhibited genetic differences in *Gbps* expression both before and after *L. major* infection (Figures 1, 2, 4 and 7; Tables 1–4). Our study extends analysis of genetic influence by Staeheli and coworkers on Gbp2b/Gbp1 expression (39), who injected forty six mouse strains by poly(I);poly(C) in order to induce interferon production and tested their spleen cells for guanylate-binding activity. Tested strains were divided into Gbp2b/Gbp1 inducible and Gbp2b/Gbp1 noninducible groups. BALB/c was in the inducible group, whereas STS, O20, and C57BL/6J belonged to noninducible one (39). Our data confirm strong genetic influence on expression of *Gbp2b/Gbp1*; however, a direct comparison of outcome of study of Staeheli et al. (39) with our results is impossible due to different experimental designs. They induced Gbp2b/Gbp1 expression by poly(I);poly(C) that is structurally similar to double-stranded RNA present in some viruses, whereas we stimulated *Gbp2b*/*Gbp1* expression by the chronic infection with parasite *L. major*.

### Comparison of Genotypes in *Gbp* Cluster on Mouse Chromosome 3 Indicates *Trans*-Regulation

Our data surprisingly showed that in several organs expressions levels of *Gbp*s in recombinant congenic strains were outside the range of their parents. In skin of uninfected mice, expression of *Gbp2b*/*Gbp1* in CcS-5, CcS-16, and CcS-20 exceeded those of both their parents BALB/c and STS (Figure 1A) and expression of *Gbp2b*/*Gbp1* in B10.O20 exceeded expression in parental strain B10 (Figure 1A). Such pattern of inheritance has been considered to be caused by *trans*-regulatory effects of non-linked or distant genes (40). The differences between parental strains and CcS/Dem strain CcS-20 persist after *L. major* infection, whereas the differences between expression of parents and CcS-5 and CcS-16 and between parent B10 and the strain B10.O20 disappear after infection (Figure 1A; Figure S1A in Supplementary Material; Tables 1A,B). Expression of *Gbp5* in skin of uninfected CcS-20 exceeded level of both parents (Figure 2A; Table 1C) but was significantly higher only than the parental strain STS after 8 weeks of infection (Figure S2A in Supplementary Material; Table 1D). CcS-5 and CcS-16 highly differed in the expression of both *Gbp1*/*Gbp2b* and *Gbp5* in lymph nodes and liver of infected mice; these strains also differed in expression of *Gbp5* in spleen (Tables 2B,D, 3D, and Tables 4B,D).

**Figure 10:**
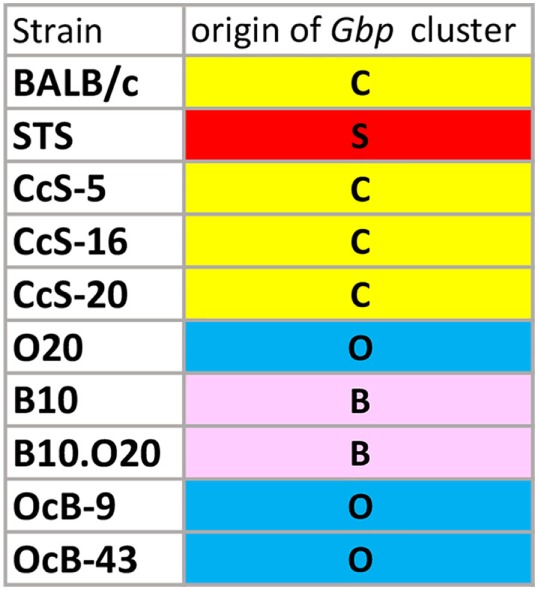
Genetic origin (alleles) of *Gbp* cluster on chromosome 3 of tested strains. C—genotype of BALB/c origin, S—genotype of STS origin, O—genotype of O20 origin, B—genotype of B10 origin.

Comparison of genotypes of the tested strains (33, 41, 42) (this study) in the *Gbp* cluster on the mouse chromosome 3 (Figure 10) revealed that strains CcS-5, CcS-16, and CcS-20 exhibiting higher expression of *Gbp* had *Gbp* genotype identical to that of BALB/c (C). Similarly, highly differing CcS-5 and CcS-16 strains carry the same *Gbp* allele. The presence of the same allele of *Gbp* gene cluster on chromosome 3 in strains that differ in other genes suggests that their differences in expression of *Gbp2*/*Gbp1* and/or *Gbp5* from one or both parents or from other RC strain are due to regulatory influence of non-*Gbp* gene(s) of STS origin carried on other genetic segments (*trans*-regulation). In the OcB/Dem series, B10.O20 carried in *Gbp* cluster B10 genotype (B), which similarly indicated *trans*-regulation of expression from O20 genome situated outside *Gbp* cluster (Table 1A; Figure 10). This *trans*-regulation can be partly overlaid by other regulatory events appearing after infection. Further genetic studies will be needed to elucidate nature of regulatory events observed in our studies.

The observations of progeny having a phenotype, which is beyond the range of the phenotype of its parents, are not rare. For example, analysis of gene expression from livers in chromosome substitution mouse strains revealed that only 438 of the 4,209 expressed genes were inside the parental range (40). These observations are due to multiple regulatory interactions, which in new combinations of these genes in recombinant congenic or chromosomal substitution strains can lead to the appearance of new phenotypes that exceed their range in parental strains.

### Increased Expression of *Gbp2b*/*Gbp1* and/or *Gbp5* in Resistant Mice Suggests Hidden Inflammation

We and others have demonstrated that *Leishmania* parasites are present not only in organs of infected susceptible mice with clinical manifestations of the disease but also in clinically asymptomatic mice of resistant strains (37, 43–46). This is also shown in Figures 3D,E and 9B,C,E. Figures 4–7 show that the expression of *Gbp2b*/*Gbp1* and/or *Gbp5* has increased after infection in at least one organ of each of the tested mice, including the resistant ones (STS, CcS-5, O20, B10, OcB-9, and OcB-43), which had no or only minimal and transient clinical symptoms. This strongly suggests that persistent parasites can contribute to the maintenance of protective immunity, which was manifested in our experiments by the increased levels of *Gbp2b/Gbp1* and *Gbp5* in resistant mice. It was demonstrated previously that this latent infection is controlled by inducible nitric oxide synthase (43) and phagocyte NADPH oxidase (46). It remains to be established, whether defense mechanisms including Gbps that were found to act against other pathogens (16, 23), operate also in *Leishmania*-infected mammalian host. In defense against *M. bovis*, Gbp2b/Gbp1 and Gbp7 could promote NADPH oxidase activity after the recruitment of gp91phox and gp22phox components to bacteria vacuoles (23), whereas parasite *T. gondii* was directly attacked via Gbp supramolecular complexes (16). The observed association (Figure 9) of Gbp2b/Gbp1 with *L. major* parasites in the skin of resistant and intermediate strains but not the highly susceptible strain BALB/c may suggest a role of this protein in response against the *L. major* pathogens.

Importantly, persistent parasites, besides stimulating protective immune reactions, can also represent a danger for hosts (45). The increased expression of *Gbp2b*/*Gbp1* and *Gbp5* in clinically asymptomatic mice reveals the price exacted from the organism by a dormant infection. This finding deserves attention, because elevated levels of human GBP1 are directly involved in the endothelial dysfunction and the regulation of endothelial progenitor cells activity in patients with the autoimmune diseases such as rheumatoid arthritis, systemic lupus erythematosus and systemic sclerosis (47). In mice, elevated levels of Gbp3 and Gbp6 were linked with the pathogenesis of atherosclerosis (48). In humans with colorectal cancer, the anti-angiogenic effect of increased levels of GPB1 was beneficial in colorectal carcinoma patients, where it was associated with sustained reduction of intratumoral angiogenic activity and improved cancer-related survival (49).

The immune reactions accompanying persistent *Leishmania* infection might be very important, because in addition to 12 million people presently suffering from the clinical manifestations of leishmaniasis (50), there are at least 120 million people with asymptomatic infection (45). It needs to be elucidated, whether such clinically asymptomatic people harboring persistent *Leishmania* parasites are more prone to immune-related diseases.

## Conclusion

Our results represent the presently most comprehensive information about expression of *Gbps* in leishmaniasis *in vivo*.

We found that expression of *Gbp2b*/*Gbp1* and *Gbp5* is under strong genetic control involving in some strains also *trans*-regulation both in uninfected and *L. major*-infected mice.

We have observed that in several organs, expression of *Gbps* in recombinant congenic strains was outside the range of their parents. Tests of different strains that carry the same *Gbp* cluster genotypes on chromosome 3 indicate a *trans*-regulation of *Gbp2b*/*Gbp1* and *Gbp5* by genes that are not closely linked to *Gbp* genes. This finding may open way to identification and manipulation of these presently unknown genes.

Our results also point out that expression of *Gbp2b*/*Gbp1* and *Gbp5* was increased even in organs of resistant mice, which might suggest a hidden inflammation. It remains to be established whether the clinically asymptomatic infection might represent danger in predisposing organism to other diseases.

Co-localization of Gbp2b/Gbp1 protein with most *L. major* parasites in skin of resistant and intermediate strains STS, CcS-5, O20, and CcS-20 but not in highly susceptible BALB/c mice suggests that this molecule might play role in defense against leishmaniasis and opens new research direction in analysis of control of persistent parasites.

## Ethics Statement

All experimental protocols utilized in this study comply with the Czech Government Requirements under the Policy of Animal Protection Law (No. 246/1992) and with the regulations of the Ministry of Agriculture of the Czech Republic (No. 207/2004), which are in agreement with all relevant European Union guidelines for work with animals and were approved by the Institutional Animal Care Committee of the Institute of Molecular Genetics AS CR and by Departmental Expert Committee for the Approval of Projects of Experiments on Animals of the Academy of Sciences of the Czech Republic (permissions Nr. 190/2010; 232/2012).

## Author Contributions

YS planned and performed parasitology and expression experiments and contributed to the writing of the manuscript. VV performed parasitology experiments, analyzed the data, and contributed to the writing of the manuscript. TK contributed to the writing of the manuscript, parasitology experiments, estimation of parasite numbers, and data analysis. HH designed and performed immunohistochemistry analysis and analyzed the data. IK contributed to the estimation of parasite numbers. MS performed parasitology experiments. PD analyzed the data and contributed to the writing of the manuscript. ML conceived the study, interpreted data, and wrote the manuscript. All authors reviewed the manuscript.

## Conflict of Interest Statement

The authors declare that the research was conducted in the absence of any commercial or financial relationships that could be construed as a potential conflict of interest.

## Abbreviations

Gbp, guanylate-binding protein (murine); GBP, guanylate-binding protein (human); *Gbp2b*/*Gbp1*, murine gene coding this guanylate-binding protein was originally named *Gbp1* and later renamed *Gbp2b*; RCS, recombinant congenic strains; CcS—BALB/c-c-STS, series of recombinant congenic containing random 12.5% of genome of the donor strain STS/A (STS) on 87.5% genome of the background strain BALB/cHeA (BALB/c); OcB—O20-c-C57BL/10-*H*-*2pz* (B10.O20/Dem), series of recombinant congenic containing random 12.5% (or 6.25% or less) of genome of the donor strain B10.O20/Dem (B10.O20) on the background strain O20/A (O20).
