# Supplementary material for: Genetic Regulation of Guanylate-Binding Proteins 2b and 5 during Leishmaniasis in Mice
Source: Front Immunol. 2018 Feb 7;9:130. doi: 10.3389/fimmu.2018.00130 (PMC5808352; doi:10.3389/fimmu.2018.00130)

SUPPLEMENTARY FIGURE 1. Differences in expression of *Gbp2b/Gbp1* in organs of infected mice.

Expression of *Gbp2b/Gbp1* in skin (A), lymph nodes (B), spleen (C) and liver (D) of 8 weeks infected female mice of strains BALB/c (n = 9 skin, 11 lymph nodes, 14 spleen, 13 liver), STS (7 skin, 6 lymph nodes, 12 spleen, 6 liver), CcS-5 (6 skin, 6 lymph nodes, 6 spleen, 6 liver), CcS-16 (6 skin, 6 lymph nodes, 6 spleen, 6 liver), CcS-20 (6 skin, 6 lymph nodes, 5 spleen, 7 liver), O20 (10 skin, 9 lymph nodes, 9 spleen, 8 liver), B10 (8 skin, 13 lymph nodes, 10 spleen, 12 liver), B10.O20 (13 skin, 11 lymph nodes, 6 spleen, 11 liver), OcB-9 (7 skin, 7 lymph nodes, 6 spleen, 7 liver) and OcB-43 (9 skin, 5 lymph nodes, 6 spleen, 6 liver) were compared. The data show the means ± SD. Only the differences between parental strains BALB/c and STS and strains of CcS/Dem series and parental strains O20 and B10 and strains of OcB/Dem series are shown. Nominal *P* values are shown.

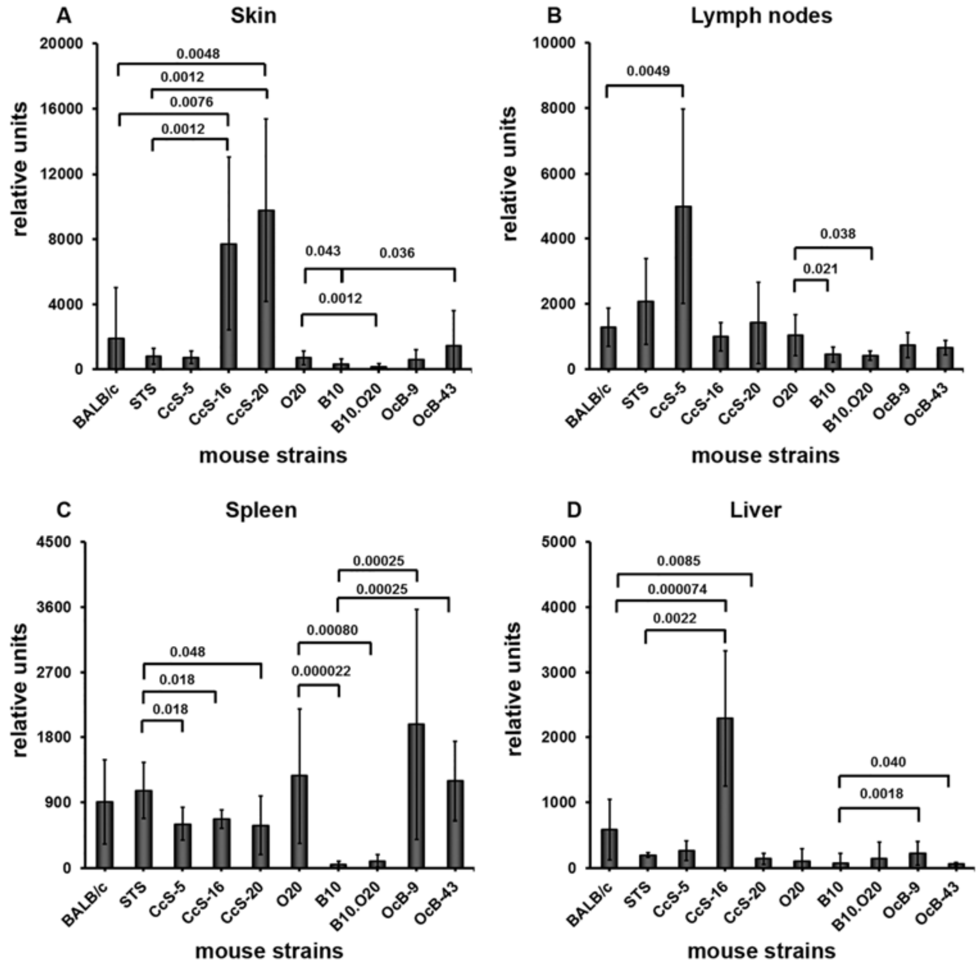

SUPPLEMENTARY FIGURE 2. Differences in expression of *Gbp5* in organs of infected mice.

Expression of *Gbp5* in skin (A), lymph nodes (B), spleen (C) and liver (D) of 8 weeks infected female mice of strains BALB/c (n = 9 skin, 11 lymph nodes, 14 spleen, 13 liver), STS (7 skin, 6 lymph nodes, 12 spleen, 6 liver), CcS-5 (6 skin, 6 lymph nodes, 6 spleen, 6 liver), CcS-16 (6 skin, 6 lymph nodes, 6 spleen, 6 liver), CcS-20 (6 skin, 6 lymph nodes, 5 spleen, 7 liver), O20 (10 skin, 9 lymph nodes, 9 spleen, 8 liver), B10 (8 skin, 13 lymph nodes, 10 spleen, 12 liver), B10.O20 (13 skin, 11 lymph nodes, 6 spleen, 11 liver), OcB-9 (7 skin, 7 lymph nodes, 6 spleen, 7 liver) and OcB-43 (9 skin, 5 lymph nodes, 6 spleen, 6 liver) were compared. The data show the means ± SD. Only the differences between parental strains BALB/c and STS and strains of CcS/Dem series and parental strains O20 and B10 and strains of OcB/Dem series are shown. Nominal *P* values are shown.

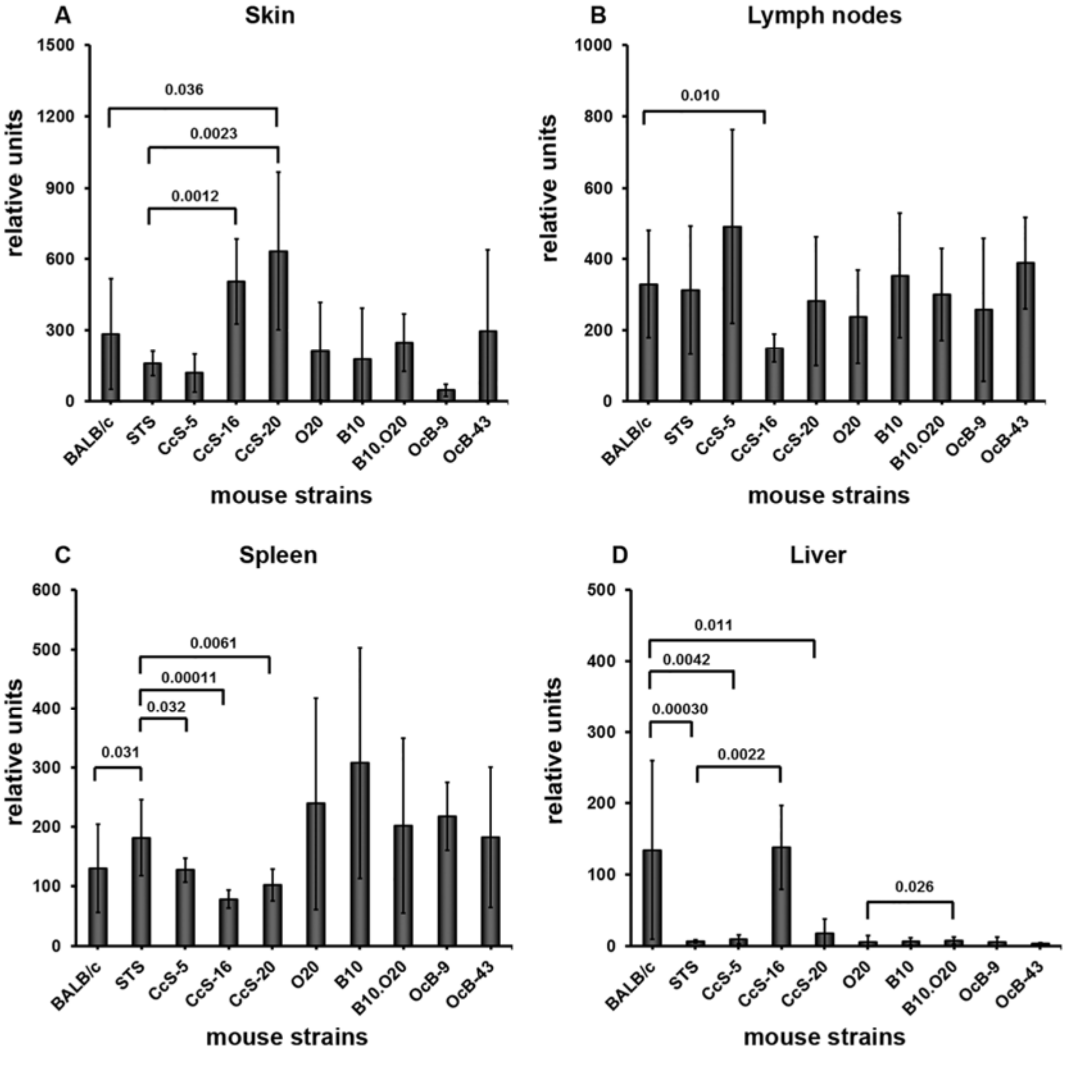

SUPPLEMENTARY FIGURE 3. Parasite load in skin of mice tested in Immunohistochemistry experiments. 48 infected female mice of strain BALB/c (n = 9), STS (n = 9), CcS-5 (n = 8), CcS-20 (n = 11), and O20 (n = 11), were tested in 2 independent experiments. The data show the means ± SD.

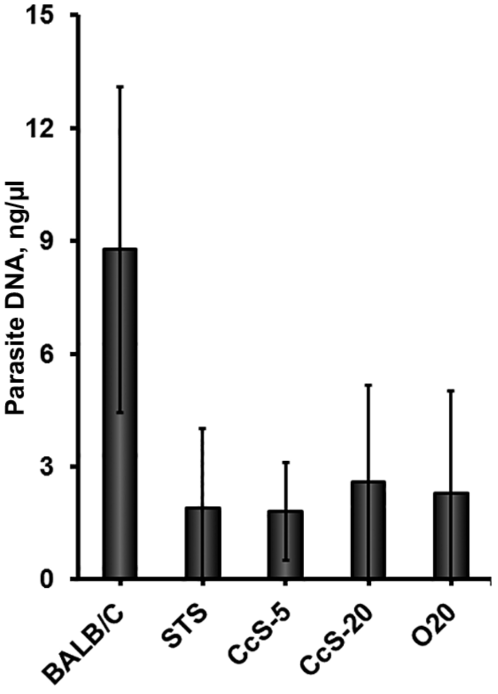

Supplement: Supplementary file 1 [file Data_Sheet_1.PDF]
